# Mapping and Summarizing the Research on AI Systems for Automating Medical History Taking and Triage: Scoping Review

**DOI:** 10.2196/53741

**Published:** 2025-02-06

**Authors:** Elin Siira, Hanna Johansson, Jens Nygren

**Affiliations:** 1 School of Health and Welfare Halmstad University Halmstad Sweden

**Keywords:** scoping review, artificial intelligence, AI, medical history taking, triage, health care, automation

## Abstract

**Background:**

The integration of artificial intelligence (AI) systems for automating medical history taking and triage can significantly enhance patient flow in health care systems. Despite the promising performance of numerous AI studies, only a limited number of these systems have been successfully integrated into routine health care practice. To elucidate how AI systems can create value in this context, it is crucial to identify the current state of knowledge, including the readiness of these systems, the facilitators of and barriers to their implementation, and the perspectives of various stakeholders involved in their development and deployment.

**Objective:**

This study aims to map and summarize empirical research on AI systems designed for automating medical history taking and triage in health care settings.

**Methods:**

The study was conducted following the framework proposed by Arksey and O’Malley and adhered to the PRISMA-ScR (Preferred Reporting Items for Systematic reviews and Meta-Analyses extension for Scoping Reviews) guidelines. A comprehensive search of 5 databases—PubMed, CINAHL, PsycINFO, Scopus, and Web of Science—was performed. A detailed protocol was established before the review to ensure methodological rigor.

**Results:**

A total of 1248 research publications were identified and screened. Of these, 86 (6.89%) met the eligibility criteria. Notably, most (n=63, 73%) studies were published between 2020 and 2022, with a significant concentration on emergency care (n=32, 37%). Other clinical contexts included radiology (n=12, 14%) and primary care (n=6, 7%). Many (n=15, 17%) studies did not specify a clinical context. Most (n=31, 36%) studies used retrospective designs, while others (n=34, 40%) did not specify their methodologies. The predominant type of AI system identified was the hybrid model (n=68, 79%), with forecasting (n=40, 47%) and recognition (n=36, 42%) being the most common tasks performed. While most (n=70, 81%) studies included patient populations, only 1 (1%) study investigated patients’ views on AI-based medical history taking and triage, and 2 (2%) studies considered health care professionals’ perspectives. Furthermore, only 6 (7%) studies validated or demonstrated AI systems in relevant clinical settings through real-time model testing, workflow implementation, clinical outcome evaluation, or integration into practice. Most (n=76, 88%) studies were concerned with the prototyping, development, or validation of AI systems. In total, 4 (5%) studies were reviews of several empirical studies conducted in different clinical settings. The facilitators and barriers to AI system implementation were categorized into 4 themes: technical aspects, contextual and cultural considerations, end-user engagement, and evaluation processes.

**Conclusions:**

This review highlights current trends, stakeholder perspectives, stages of innovation development, and key influencing factors related to implementing AI systems in health care. The identified literature gaps regarding stakeholder perspectives and the limited research on AI systems for automating medical history taking and triage indicate significant opportunities for further investigation and development in this evolving field.

## Introduction

### Background

In health care, efficient management of the flow of patients through the health care system is important to ensure that care is accessible and appropriate, given the needs of patients [1]. With the introduction of artificial intelligence (AI) systems, there has been growing interest in using these technologies to improve patient flow [2]. Research in this area remains in its early stages, constituting a disjointed field [3]. In addition, AI systems hold great promise to make patient flows more efficient at the admission of patients by improving triage [4,5].

Traditionally, triage involves a health care professional taking the patient’s medical history to systematically decide the optimal prioritization and assess the appropriate treatment for the patient. There are different types of triage, each with its distinctive elements, such as inpatient triage, emergency department triage, incident triage, military triage, and mass casualty triage, all of which possess the aforementioned characteristics [6]. AI systems could potentially increase the efficiency of triage processes by replacing health care professionals in taking the patient’s medical history and directing patients to the most appropriate treatment. This could effectively reduce health care professionals’ workload and allow for a more optimal allocation of their time [4,5]. Despite the high hopes and AI systems showing good performance in studies, only a small number have been implemented in operational health care systems [7,8].

To fully understand how AI systems for automating medical history taking and triage could be used to create value in the health care context, it is important to understand the entire development and implementation process, including data collection, algorithm development, model validation, and real-world implementation [9]. Each of these steps presents unique challenges and considerations that must be carefully addressed to ensure successful integration into health care. It is essential to determine at which stage of the innovation development process the AI systems are halted or hindered before they are integrated into health care practices [7,8]. Many technical and nontechnical factors affect the implementation process. Technical challenges, such as data quality and interoperability, algorithm accuracy, and system reliability, must be carefully evaluated and addressed [9-11]. In addition, ethical considerations, regulatory frameworks, organizational barriers, and patient acceptance play critical roles in the implementation and use of these technologies [10]. By studying these factors, we can identify the barriers that hinder the effective implementation of AI systems and facilitate strategies to overcome them.

However, it is equally important to recognize that the successful implementation of AI systems in health care extends beyond the technical and regulatory aspects. The perspectives of a wide range of stakeholders must be considered as they can offer valuable insights and contribute to the overall effectiveness and acceptance of these systems [12,13]. Stakeholders include health care providers, patients, decision makers, regulatory bodies, administrators, and other context-based relevant actors. Each stakeholder group brings unique perspectives, priorities, and concerns, which must be integrated into the development and deployment of AI systems for medical history taking and triage [12,13]. By incorporating such diverse perspectives, we can ensure that these technologies align with the needs, values, and expectations of the stakeholders they seek to serve.

In light of these considerations, it is crucial to conduct a scoping review to map and summarize empirical research on AI systems for automating medical history taking and triage. By systematically reviewing the literature, we can identify the current state of knowledge across various stages of the innovation development process, gain insights into the hindering and facilitating factors affecting the implementation and use of AI systems, and consider the perspectives of different stakeholders. Such a comprehensive review will not only help identify gaps in knowledge but also guide future research efforts and inform evidence-based practice for integrating AI systems for automating medical history taking and triage in health care settings.

### Aim and Research Questions

The aim of the scoping review was to map and summarize empirical research on AI systems for automating medical history taking and triage. The following research questions (RQs) guided this review:

1. What are the characteristics of research publications on AI systems for automated medical history taking and triage in health care?
2. Whose perspective (researcher, health care professional, or patient) of the AI systems is described?
3. At which stage of the innovation development process are the AI systems studied?
4. What facilitating factors and barriers are considered in relation to the introduction of the AI systems?

## Methods

### Study Design

The scoping review was designed according to the framework of Arksey and O’Malley [14], which has been widely used to explore, summarize, and draw conclusions about the overall state of research on new technology and health care (refer to the studies by Sharma et al [15], Gama et al [16], and Wikström et al [17]). The characteristics of included research publications (RQ1) and whose perspective is described in the publications (RQ2) are questions commonly addressed in scoping reviews. To investigate at which stages of the innovation development process the AI systems in the research publications were studied (RQ3), we adopted the Technology Readiness Level (TRL) scale for clinical readiness by Fleuren et al [7]. The scale is based on the original TRL scale developed by the National Aeronautics and Space Administration to evaluate technological maturity. It measures the clinical applicability of AI (ie, machine learning) systems on a scale of 1 to 9 (1 being the least mature and 9 being the most mature) [7,18]. To identify and report patterns in facilitating factors and barriers to the introduction of AI systems (RQ4), we used techniques from thematic analysis (ie, we coded the data and generated themes) [19]. The scoping review was reported according to the PRISMA-ScR (Preferred Reporting Items for Systematic reviews and Meta-Analyses extension for Scoping Reviews) guidelines [20] (Multimedia Appendix 1), and a scoping review protocol was established before conducting the review.

### Search Strategy

To cover any literature relevant to the scope of the review, we used 4 key terms: *health care*, *AI systems*, *medical history-taking*, and *triage* (Textbox 1). To systematically capture literature related to the key terms and synonyms, they were searched using both index terms (ie, subject headings) and free text, as outlined in Multimedia Appendix 2. To generate an extensive coverage of relevant studies, as recommended in scoping reviews [14], 5 major databases for health care–related research were searched: PubMed, CINAHL, PsycINFO, Scopus, and Web of Science Core Collection. The search was conducted in October 2022. All the databases were searched for studies published between January 2000 and September 2022. The search strings were first piloted in one of the databases (PubMed) and then further developed with the support of a research librarian before systematically searching all 5 databases. Textbox 2 provides an illustration of one of the search strings.

**Key terms used in search and their definitions.**

- Healthcare: “...all the organizations, institutions and resources that are devoted to producing health actions” [21]
- Artificial intelligence (AI) systems: “...a machine-based system that can, for a given set of human-defined objectives, make predictions, recommendations, or decisions influencing real or virtual environments. AI systems are designed to operate with varying levels of autonomy.” [22]
- Medical history-taking: The process of acquiring information from a patient on medical conditions and past treatment [23,24]
- Triage: The process upon which the optimal prioritization and assessment of the appropriate next step for the patient can be decided [6]

**Search string used for the PubMed database (publications were filtered for publication date from January 1, 2000, to September 30, 2022).**

((“neural networks, computer”[MeSH Terms:noexp] OR “artificial intelligence”[MeSH Terms:noexp] OR “deep learning”[MeSH Terms] OR “supervised machine learning”[MeSH Terms] OR “artificial intelligence”[Title/Abstract] OR “deep learning”[Title/Abstract] OR “supervised machine learning”[Title/Abstract]) AND (“delivery of health care”[MeSH Terms] OR “health care”[Title/Abstract] OR “healthcare”[Title/Abstract] OR “medical”[Title/Abstract] OR “clinical”[Title/Abstract])) AND (“Medical History Taking”[MeSH Terms] OR “anamnes*”[All Fields] OR “Triage”[MeSH Terms])

### Eligibility Criteria

The eligibility criteria were set to identify research publications that presented empirical studies or systematically conducted reviews of empirical studies that focused on AI systems for automating medical history taking and triage in health care. A retrieved study was eligible for inclusion if it (1) contained a title and abstract; (2) was written in English; (3) was published between January 1, 2000, and September 30, 2022; (4) was peer reviewed; (5) analyzed empirical data or systematically reviewed empirical data; and focused on (6) health care, (7) AI, and (8) medical history taking or triage (Textbox 3). Before using the eligibility criteria for study selection in all databases, we piloted the criteria for the studies retrieved from one of the databases (PubMed). The criteria were not altered after piloting.

**Eligibility criteria.**

**Inclusion criteria**

Contained title and abstract: Yes

English language: Yes

Publication date: January 1, 2000, to September 30, 2022

Peer reviewed: Yes

Empirical study: Analyzed empirical data or systematically reviewed articles with empirical data

Focused on health care: Yes

Focused on artificial intelligence: Yes

Focused on medical history taking or triage: Yes

**Exclusion criteria**

Contained title and abstract: No

English language: No

Publication date: Before December 31, 1999

Peer reviewed: No

Empirical study: Analyzed based on theoretical or hypothetical reasoning

Focused on health care: No

Focused on artificial intelligence: No

Focused on medical history taking or triage: No

### Study Selection

First, studies identified through the search strategy were imported into Endnote software (version 20; Clarivate) and exported to the Rayyan web application (Rayyan Systems, Inc) [25] for controlled removal of duplicates. After the removal of duplicates, 2 of the authors (ES and HJ) independently screened the retrieved titles and abstracts for checking their eligibility for inclusion. When screening studies for inclusion, the authors checked the retrieved studies for inclusion according to the inclusion criteria in chronological order, that is, if criterion 1 was met, the authors checked if the retrieved study met inclusion criterion 2, and so on. Conference proceedings were excluded because they are often preliminary and based on limited analyses [26]. Editorials, commentaries, viewpoints, discussion and opinion papers, and book chapters were excluded because they often do not undergo a rigorous and impartial peer-review process. Narrative and integrative reviews were excluded because they did not systematically analyze or review empirical data. Any disagreements during the screening of titles and abstracts were discussed with a third author (JN) until a consensus was reached. Full texts of the studies that met the inclusion criteria after screening titles and abstracts were assessed for eligibility. ES and HJ independently screened the full texts of the studies. Disagreements concerning the full-text screening were discussed among all the authors until a consensus was reached.

### Data Extraction

A data extraction template was developed by one of the authors (ES) based on the RQs. The template was piloted with 5 studies before extracting the data from all included research publications. The template was not altered after piloting. To extract data relevant to the RQs, the template contained columns for information on the characteristics of the research publications (RQ1; authors, publication year, country, clinical setting, sample population, study aim, study design, type of AI system, and main task performed by AI system), the perspective described in the research publications (RQ2; ie, the researchers’, health care professionals’, or patients’ perspectives), the TRLs (1-9) of the AI systems in the research (RQ3), and facilitators and barriers to the introduction of the AI systems (RQ4). To identify the type of AI system and the main task performed by the AI system (RQ1), we adopted the operational terms presented in the Organisation for Economic Co-operation and Development Framework for the Classification of AI systems [27].

We extracted data in relation to RQs 1 to 3 from all included studies, whereas data in relation to RQ4 were extracted only from the studies deemed to have TRLs equal to 6 or higher (ie, they reported a demonstration of an AI system in a real health care environment) and for literature reviews. In total, 2 (3%) of the 77 studies with TRLs ≥6 [28,29] and 2 (33%) of the 6 literature reviews did not report the introduction of AI systems in real health care environments [30,31]. Consequently, data from these studies did not address RQ4.

Data on the characteristics of the research publications (RQ1) were extracted from the entire article; data on the perspective described in the research publications (RQ2) and the TRLs (RQ3) were extracted from the methods, results, and discussion sections of the articles. Finally, data on facilitators and barriers to the introduction of the AI systems (RQ4) were extracted from the results, discussion, and conclusions sections of the research publications.

### Data Analysis

Following the framework proposed by Arksey and O’Malley [14], we began the analysis with a basic numerical analysis of the extracted data and produced tables to report the distribution of the characteristics of the studies (RQ1), the perspective described in the studies (RQ2), and their TRLs (RQ3). Thereafter, to provide a narrative account of the facilitating factors and barriers to the introduction of the AI systems (RQ4), we coded and organized the extracted data thematically [19] This involved an inductive iterative process in which we coded the extracted text on the facilitating factors and barriers and collated the codes into potential themes, which we then revised and refined. ES and HJ independently coded the data, compiled all the codes, and developed the final themes. The thematic analysis was finalized through a group discussion among all 3 authors.

## Results

### Overview

**Figure 1:**
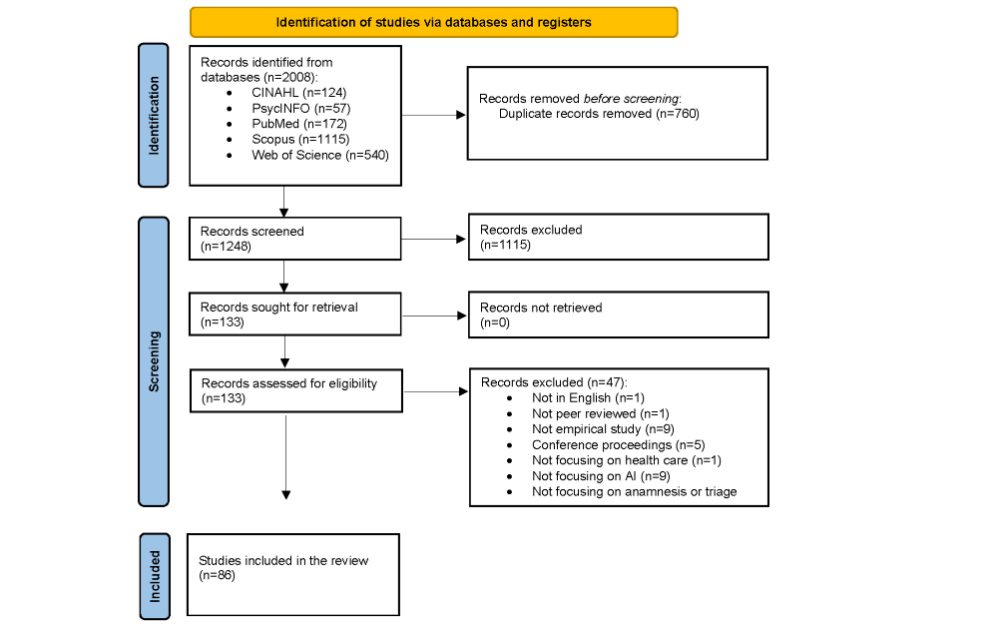
PRISMA (Preferred Reporting Items for Systematic Reviews and Meta-Analyses) flowchart. AI: artificial intelligence.

A total of 2008 records were retrieved from the electronic databases. After removing duplicates (n=760, 37.85%), 1248 records remained and were included in the screening. Of these, 133 (10.66%) records were assessed for eligibility in full text, and 86 (64.7%) were deemed eligible for inclusion. The PRISMA (Preferred Reporting Items for Systematic reviews and Meta-Analyses) flow diagram gives the details on the identification and screening of studies (Figure 1) [32].

### RQ1: Characteristics of the Research Publications

The included studies were published between 2000 and 2022, but most (63/86, 73%) of the studies were published between 2020 and 2022. The remaining studies (16/86, 19%) were published predominantly between 2018 and 2019. Only a small number of studies (7/86, 8%) were published between 2000 and 2017. Most of the studies were from Asia (26/86, 30%), particularly China (8/86, 9%); North America (25/86, 29%), predominantly the United States (23/86, 27%); and Europe (22/86, 26%), primarily the United Kingdom (9/86, 10%). A small number of studies (13/86, 15%) were from Africa (1/86, 1%), Oceania (5/86, 6%), and South America (4/86, 5%) or did not specify the country of origin (3/86, 3%). The most common clinical contexts in focus in the included studies were emergency care (32/86, 37%), followed by radiology (12/86, 14%) and primary care (6/86, 7%). In addition, a large number of studies (15/86, 17%) did not specify the clinical context in focus. The sample population in most studies consisted of patients (70/86, 81%), which included datasets of different types of patient records. In the remaining studies, the sample population was health care professionals (9/86, 10%), citizens (2/86, 2%), or unspecified (5/86, 6%). In these studies, the sample size was small, and there was a higher degree of involvement of the participants in the study. Regarding study design, a large number of studies were retrospective studies (31/86, 36%) or did not specify any specific design (29/86, 34%). Other study designs were, for example, prospective studies (7/86, 8%), different types of pilot studies (4/86, 5%), or reviews (4/86, 5%). The most common type of AI system was hybrid models (68/86, 79%), that is, they combined statistical and symbolic approaches. Most (40/86, 47%) of the AI systems studied were used for forecasting, that is, they predicted future outcomes based on past and present behavior or recognition (36/86, 42%), which involved identifying and categorizing data into specific classifications or performing image segmentation and object recognition. Definitions of the types of AI systems and their main tasks used in this review can be found in the Organisation for Economic Co-operation and Development Framework for the Classification of AI systems [27]. Multimedia Appendix 3 provides an overview of the characteristics of all the 86 included studies [28-31,33-114].

### RQ2: Perspectives Described in the Publications (Among Researchers, Health Care Professionals, or Patients)

Although the sample population in a large number of the included studies consisted of patients, these studies did not portray their perspectives. In most (83/86, 97%) of the included studies, researchers’ perspectives were portrayed (ie, researchers’ description and interpretation of the AI system and the results of the particular study. Only 1 (1%) study reflected the patients’ perspectives [87]. It described patient ratings of the usability and interface of an AI system for medical history taking in an emergency department. The same study also portrayed physicians’ and 10 nurses’ ratings for the same AI system. Overall, patients, physicians, and nurses were strongly positive about the AI system as a support for patient-clinician communication [87]. Another study described health care professionals’ perspectives [63]. More specifically, it explored how emergency triage nurses understood, contextualized, and incorporated an AI-based decision support system into their work on triaging patients. Initially, nurses expressed apprehension that the AI system lacked the cultural and contextual understanding necessary for patient triage. However, they later discovered that the system helped them provide safe care [63]. In total, 2% (2/86) of the studies presented health care professionals’ perspectives [63,87]. Additional studies were conducted using health care professionals as the sample population. However, these studies focused solely on health are professionals’ decision-making performance in relation to triage with AI systems, without providing insights into their perspectives [40,44,49,59,77,80,95]. In addition, 1% (1/86) of the studies portrayed citizens’ (ie, men and women aged 18-80 y) perspectives on an AI system with a conversational user interface for self-guided medical history taking [46]. The citizens completed a usability test and questionnaire concerning the usability, design, and functionality of the AI system. Overall, the citizens stated that they felt comfortable with the system; however, the authors noted that the level of technical competencies and educational levels were high among the participants, limiting the generalization to patient groups [46].

### RQ3: Stages of the Innovation Development Process (TRLs)

Most (76/86, 88%) of the included studies did not demonstrate or validate AI systems for medical history taking and triage in clinical environments (ie, they were concerned with clinical problem identification; proposal of a model or solution; or model prototyping, development, or validation. These studies scored ≤5 on the TRL scale for clinical readiness [7]. A few (6/86, 7%) studies scored ≥6 on the TRL scale (ie, they described real-time testing of AI models, workflow implementation, evaluation of clinical outcomes, or model integration). In total, 5% (4/86) of the studies, which were reviews of several different empirical studies, were not considered applicable to the TRL scale [30,31,52,57]. An overview of the included studies and their TRL scores is presented in Table 1.

**Table 1:** Technology Readiness Level (TRL) by Fleuren et al [7] of the included studies (n=82).

TRLs	Clinical definitions	Studies, n (%)
1	Clinical problem identification	0 (0)
2	Proposal of model or solution	0 (0)
3	Model prototyping	8 (9)
4	Model development	50 (58)
5	Model validation	18 (21)
6	Real-time model testing	1 (1)
7	Workflow implementation	2 (2)
8	Clinical outcome evaluation	1 (1)
9	Model integration	2 (2)

No studies were classified as TRL 1 or 2. A small number (8/86, 9%) of studies achieved TRL 3, indicating prototyping or model development of an AI system [46,82,86,93,98,103,105,108]. Examples of studies in this category include an AI-based field triage tool [82], triage of medical referrals [105], and a self-diagnosis system for emergency dental care [93]. A substantial number (50/86, 58%) of studies achieved TRL 4, that is, they demonstrated the potential of AI systems or optimized and validated these using clinical data. Examples of these studies include triage of adult chest radiographs [36], triage using digitalized patient histories in primary care [49], and triage of patients with COVID-19 under limited health care resources [69]. Several (18/86, 21%) studies were categorized as TRL 5. This means that they validated AI models using realistic datasets other than the population used to train or test the AI model. This particular type of validation data was either retrospective [48] or prospective [40]. One study attained a TRL of 6; it investigated the testing of an AI model in real time, specifically, an algorithm for automated diabetic retinopathy screening [28]. In addition, 2% (2/86) of the studies examined AI models integrated into clinical workflows and assessed their outcomes, resulting in an assessment of TRL 7 [29,87]. The studies had a common theme of emergency care and focused on a symptom-taking tool [87] and a rapid and laboratory-free COVID-19 triage [29], respectively. One study achieved a score of 8 on the TRL scale, that is, it evaluated the clinical outcomes of an implemented AI model. This study examined a medical history–taking system that generated differential diagnoses in an outpatient department [65]. In total, 2% (2/86) of the studies focused on the postimplementation phase of AI systems and thus achieved the TRL 9 [63,81]. One study focused on the impact of cultural embeddedness when implementing an AI system for emergency care triage [63]. Another study investigated the user demographics and levels of triage acuity of a symptom checker chatbot used within a large integrated health system [81].

### RQ4: Facilitating Factors and Barriers to the Introduction of the AI Systems

From the 6 retrieved studies that reported the introduction of AI systems for automating medical history taking and triage in health care, we identified 4 themes relating to facilitating factors and barriers to their introduction [52,57,63,65,81,87]. The themes were related to technical aspects, contextual and cultural factors, end users, and evaluation (Table 2). The theme denoted as *technical aspects* pertained to the AI systems’ design, integration, requirements, and performance. The theme of *contextual and cultural* factors was associated with the organization and patient populations in which the AI systems were introduced. The theme *end users* encompassed the viewpoints and experiences of patients and health care professionals with regard to AI systems. The theme *evaluation and regulation* covered various aspects of evaluating, regulating, and setting guidelines for AI systems.

**Table 2:** Facilitating factors and barriers to the introduction of artificial intelligence (AI) systems.

	Facilitating factors	Barriers
Technical aspects	Flexible design of AI system [52] Integration with existing technical systems [87]	Requirements of AI system [52] Insufficient performance of AI systems [63,65,87] Lack of integration with existing technical systems [57]
Context and culture	Understanding of a setting’s organizational context, clinical workload, and cultural competence [63]	Delays in the start-up of AI systems [57] Highly mixed patient populations, which was a challenge for the AI system [87]
End users	Consideration of patient’s perspectives [57] Adequate training and the possibility to understand the AI system [52,63,81] Innovative methods for promoting use among health care professionals [57] Communication of benefits of using the AI system [52,63]	Lack the acceptance of diagnosis provided by the AI system [65] Negative receptivity among health care professionals [63] Health care professionals’ lack of time [65] Low comprehensiveness among specific patient groups [87] Lack of eHealth literacy among patients [57,87] Insufficient language skills among patients [87] Low number of users [57] Lack of opportunities for patients to learn how to use the system [87]
Evaluation	Formulation of evaluation guidelines and regulations [57] Initial and ingoing and evaluation [57] User-focused evaluation [87]	Lack of regulation and guidelines for evaluation [57] Lack of external validation [52]

Of the 6 studies, 5 (80%) reported factors related to technical aspects of the introduction of AI systems for automating medical history taking and triage that either facilitated or hindered its introduction into clinical settings [52,57,63,65,87]. Technical facilitators included a flexible design that allows the AI system to be implemented in different geographical areas [52]. Furthermore, doctors and nurses perceived that integrating AI systems with electronic health records would enhance their usefulness [87]. Technical barriers involved the amount of information required by AI systems, which might not be possible to collect in the short time required for the triage process in emergency departments [52]. An additional issue to consider was the inadequate performance of AI systems. This was due to their lack of cultural understanding of the context and the patient population they served [63], limited accuracy, particularly when dealing with patients with complex presentations, such as older adults [65], and the absence of optimization for certain patient groups, such as those with neurological symptoms [87]. The lack of integration with established health care systems constituted an additional barrier associated with the technical attributes of the AI systems [57].

A total of 3 (50%) of the 6 studies reported facilitating barriers related to context and culture [57,63,87]. Facilitating factors included greater integration of the AI system into the clinical workflow, as nurses and physicians believed this would enhance its usefulness [87]. In addition, the knowledge of the organizational context, clinical workload and culture, skills, and recognized behavior change techniques should be considered when implementing AI systems. Contextual and cultural barriers included delays in the start-up of AI systems due to strategic decision-making processes [57] and the use of these systems in settings with highly mixed patient populations, such as emergency departments. This posed a challenge for AI systems, as reported by physicians, ultimately impacting their usefulness [87].

Of the 6 studies, 5 (80%) reported facilitating factors and barriers in relation to end users [52,57,63,81,87]. The consideration of patients’ perspectives in adapting the AI system for accessibility and usefulness as well as innovative approaches to promote use among health care professionals, such as having superusers [57], were identified as facilitating factors. Other factors that facilitated the introduction of AI systems for automating medical history taking and triage included providing adequate training for health care professionals, as this is crucial given the systems’ dependence on their users, and enabling them to effectively use the system [52,63,81] while also supporting patients through familiar health delivery mechanisms [81]. In addition, effective communication regarding the potential benefits of AI systems in enhancing patient care, resource use [52], and patient outcomes [63] was found to facilitate their adoption. Notably, the significance of the latter aspect was emphasized for its widespread uptake [63]. End-user barriers to the uptake of the AI system included physicians’ nonacceptance of AI-generated diagnosis [65], negative receptivity toward rapid implementation [63], and health care professionals constrained by limited time to use the AI system [87]. In addition, a lack of comprehension among certain patient groups (such as those with neurological symptoms), inadequate eHealth literacy [57,87], linguistic deficiencies in patients (specifically, in expressing complaints in a way that the AI system can understand) [87], and limited opportunities for patients to acquire the necessary skills to use AI tools, due to limited occasions for using it [57,87], were additional barriers linked to end users.

Facilitating factors and barriers related to evaluation were reported in 50% (3/6) of the studies [52,57,87]. Factors suggested to facilitate the introduction of AI systems were the formulation and implementation of evaluation guidelines and regulations to ease the development and use of AI systems [57]. Initial and ongoing assessments of AI systems and evaluations focused on users were facilitating factors for assessing the safety and effectiveness of introducing AI systems in complex environments [87]. Barriers related to evaluation included the current lack of evaluation regulations and guidelines [57] as well as the validation of AI systems [52].

## Discussion

### Principal Findings

The goal of this study was to analyze and compile existing empirical studies on AI systems used for automating medical history taking and triage in health care. This review shows that research on AI systems to automate medical history taking and triage in clinical settings is still at an early stage, articulating a large gap to be bridged between model development and the bedside, similar to what has been highlighted in previous research on AI in health care [7,15,16,115,116]. There is a paucity of studies demonstrating AI systems to automate medical history taking and triage in clinical settings, representing the perspectives of patients or health care professionals, and providing information on facilitators and barriers to the introduction of these AI systems. The findings on the state of research in this area should be understood in light of some of the characteristics of the included research studies, indicating that research on AI systems to automate medical history taking and triage in health care remains relatively new and underdeveloped, and is mainly conducted in a nonclinical research environment of prototyping, development, and validation. While it is crucial to establish a solid research foundation that tests and validates AI systems before their implementation in clinical settings, which could potentially impact care quality, safety, clinical outcomes, and working conditions for health care professionals, it is also essential to conduct research that puts these systems into practical use to showcase their potential [117]. However, the current level of research on AI systems for automating medical history taking and triage remains inadequate.

### Characteristics of the Research Publications

The study revealed a notable surge in research on AI systems for automating medical history taking and triage over the past 3 years, indicating a growing interest in leveraging AI technologies in relation to managing patient flows in health care settings. Geographically, research was predominantly concentrated in Asia, North America, and Europe, aligning with regions known for both advanced health care infrastructure and research capabilities. The prevalence of studies focusing on emergency and primary care underscores the potential of AI systems to automate medical history taking and triage in first-line care, which globally faces challenges in meeting the needs of the people it is intended to serve [118,119]. Many (12/86, 14%) studies also focused on radiology, illustrating the potential of AI systems to assist radiologists in image triage, a finding that is not surprising, given the progress of AI in image recognition tasks [120]. However, a significant (15/86, 17%) proportion of studies did not specify the clinical context, suggesting the need for more context-specific investigations to better understand the applicability of AI systems for medical history taking and triage. A large number (31/86, 36%) of studies also had a retrospective design, which, together with the large group of non-context–specific studies, may indicate a trend toward horizontal research rather than toward the implementation of AI systems for automating medical history taking and triage in clinical settings. As previous studies have suggested, this type of research and development may be partly due to the challenges of conducting studies that actually implement AI in real clinical settings [116]. The most common type of AI system was hybrid models that performed tasks related to forecasting or recognition. These findings resonate with previous research on the ways in which machine learning has been used to predict the flow of patients through health care systems [3]. Most (70/86, 81%) studies used patient datasets as their sample population, comprising patient records that were used to test, validate, and prototype AI models. This also meant that they focused on the technical capabilities of AI systems to automate medical history taking and triage based on a specific set of patient records, rather than their usability for health care professionals or patients.

### Perspectives Described in the Publications (Among Researchers, Health Care Professionals, or Patients)

While the promise of AI systems for automating medical history taking and triage to transform health care is well recognized, this study highlights an imbalance in stakeholder perspectives in the current research. Researchers’ viewpoints overwhelmingly dominated the literature. Despite providing insights into the description and interpretation of AI systems designed for medical history taking and triage, there has been limited exploration of the critical viewpoints stemming from patients and health care professionals regarding their practical application in clinical settings. Previous research has argued that the imbalance between different stakeholder groups’ perspectives in research on AI in health care should be addressed to meet the distinct needs of different groups [121]. Furthermore, the importance of the acceptability of interventions in routine health care practice is widely recognized in the fields of intervention, innovation, implementation, and improvement science [117]. Therefore, it is essential to consider the views of patients and health care professionals regarding the use of AI systems to automate medical history taking and triage. This will help to ensure the successful implementation of these systems. The positive feedback from patients on usability and support in patient-clinician communication from AI systems for automating medical history taking and triage emphasizes the potential benefits of involving patients in the design and evaluation of such systems. Previous research investigating a rule-based, automated medical history–taking app [122] even deemed patient involvement in the development process of these types of tools necessary. One additional study delved into health care professionals’ perspectives, focusing on concerns and adjustments when integrating AI systems for automating medical history taking and triage into the triaging processes. The study found that nurses were uneasy with the AI system’s acontextual nature [63]. To increase trust in AI systems, researchers suggest that focusing solely on technical aspects, such as objectivity, efficiency, and accuracy, is insufficient. AI systems must also be aligned with the values and principles of the clinical environment in which they are used. This is crucial because people are more inclined to trust and embrace AI systems when they perceive them as aligning with the inherent value, significance, and utility of the context in which they are deployed [123].

### Stages of the Innovation Development Process: TRLs

The application of the TRL framework [7] provided a structured understanding of AI systems for automating medical history taking and triage development stages. Most (76/86, 88%) studies focused on lower TRLs, which aligns with the prevalence of studies that emphasize developmental work and potential demonstrations in the broader field of applying AI in health care [15]. This disparity underscores a critical gap in our understanding. This highlights the pressing need for more comprehensive investigations into the practical implementation and validation of AI systems in real health care settings. These higher TRL evaluations are pivotal in ensuring that AI technologies not only show promise in controlled environments but also prove their effectiveness and reliability when deployed in complex and dynamic safety-critical settings in health care practice [124]. Such research can contribute significantly to building trust and confidence in AI systems within the medical domain and promoting their implementation and use in practice.

### Facilitating Factors and Barriers to the Introduction of AI Systems

The introduction of AI systems for automating medical history taking and triage is a complex process influenced by several factors, both hindering and facilitating its implementation. One set of barriers described lies in technical aspects, encompassing system performance and integration. The effectiveness and seamless integration of these AI systems into existing health care workflows will significantly impact their adoption and use. In addition to the technical issues, contextual and cultural factors are also important. The organizational context within health care settings as well as the diversity of patient populations shaped adoption. Different health care institutions, stakeholder roles, and patient perspectives may have unique requirements and expectations regarding AI integration in health care [9,125]. Recognizing the importance of stakeholder and patient perspectives highlights the need for tailoring AI systems to cater to context- and situation-specific needs and concerns [9,126,127]. Regulatory and evaluative considerations add another layer of complexity to the integration process. The development of guidelines and user-focused evaluations is essential to ensure the safety, efficacy, and ethical use of AI systems in health care [128-130]. These considerations underscore the need for a well-structured and carefully monitored approach to the implementation of AI in medical history taking and triage.

### Limitations

The study’s strengths include a thorough review of records with rigorous screening. The search strategy covered 5 electronic databases, ensuring comprehensiveness. However, it missed the opportunity to explore gray literature, which could have revealed additional cases that may present ongoing or completed work not yet in the research literature. We decided not to include conference proceedings in this review due to their often preliminary nature and limited analysis [26]. Upon examining the conference papers identified in the search, it became evident that they represented a diverse array of formats and scopes and varied significantly in how they were quality assessed and made accessible. This heterogeneity posed a challenge for their integration into a pooled analysis alongside more uniform and rigorously peer-reviewed papers. Nevertheless, in instances where the evidence is limited or contradictory, which is often the case in research on AI, the incorporation of conference proceedings can prove advantageous for systematic reviews [26]. Therefore, it is recommended that future reviews in this domain of research consider the incorporation of high-quality conference proceedings. We did not manually search the reference lists of the included studies to add more content to the search because an as stringent as possible review of the current literature was sought. Identifying studies focusing on AI systems can be a complex task due to the lack of consensus on their definition. To ensure clarity for the reader, we have aimed to be transparent about how we define AI systems in this review. To maintain clarity, we based our inclusion on how the original authors described their systems as AI, relying on their accuracy. Because AI definitions vary, this introduces subjectivity, potentially affecting the consistency and comparability of the included articles. We suggest that future research consider this challenge in greater depth. We used a search strategy similar to that used in previous scoping reviews to identify AI systems [15,16]. The studies reviewed in this study concentrate on medical history taking and triage, both crucial processes for managing patient flow by assessing a patient’s condition and referring them to the appropriate care. However, it is important to note that this approach may have some limitations. Therefore, we recommend that future research conduct separate literature reviews for each of these patient flow management processes. The eligibility criteria for study publication dates were set to identify any early studies on the topic of this review, despite AI applications in health care being a more recent phenomenon [131]. To ensure consistency, all full-text screening decisions were confirmed in pairs and discussed at biweekly meetings. This approach encouraged open discussions to address doubts, exclusion criteria, and differing interpretations. Conflicting interpretations and uncertainties were resolved through discussion.

### Conclusions

This scoping review contributes to the understanding of AI system implementation in health care by providing insights into current trends, stakeholder perspectives, innovation development stages, and influencing factors. It highlights gaps in patient perspectives and the underrepresentation of higher TRLs, indicating opportunities for further research. To realize the full potential of AI systems for automating medical history taking and triage in health care settings, it is crucial to integrate patients’ voices into AI development, enhance real-world validation, and address the multifaceted challenges highlighted by end users and regulations. As health care systems evolve, embracing AI technologies while ensuring alignment with clinical and patient needs remains a key challenge for future research and implementation efforts. These multifaceted factors collectively shape the landscape of AI system adoption in the context of automating medical history taking and triage. Therefore, a holistic and inclusive approach is necessary to ensure the effectiveness and ethical use of these types of AI systems.

## Appendix

Multimedia Appendix 1 PRISMA-ScR (Preferred Reporting Items for Systematic Reviews and Meta-Analyses extension for Scoping Reviews) checklist.

Multimedia Appendix 2 Search strategy.

Multimedia Appendix 3 Characteristics of the included studies.

## Abbreviations

AI: artificial intelligence
PRISMA: Preferred Reporting Items for Systematic reviews and Meta-Analyses
PRISMA-ScR: Preferred Reporting Items for Systematic reviews and Meta-Analyses extension for Scoping Reviews
RQ: research question
TRL: Technology Readiness Level

## Data Availability

All data generated or analyzed during this study are included in this published article and its supplementary information files.
