# Supplementary material for: Mapping and Summarizing the Research on AI Systems for Automating Medical History Taking and Triage: Scoping Review
Source: J Med Internet Res. 2025 Feb 6;27:e53741. doi: 10.2196/53741 (PMC11843066; doi:10.2196/53741)
Supplement: Multimedia Appendix 2 [file jmir_v27i1e53741_app2.docx]

## **Multimedia Appendix 1**

Table 1. Search blocks by database (PubMed, CINAHL, PsycINFO, Scopus, Web of Science Core Collection)

| **DATABASE** | **SEARCH BLOCKS** | | |
| --- | --- | --- | --- |
|  | **Artificial intelligence (AI)** | **Healthcare** | **Medical history-taking and/or triage** |
| **PubMed** | ("neural networks, computer"[MeSH Terms:noexp] OR "artificial intelligence"[MeSH Terms:noexp] OR "deep learning"[MeSH Terms] OR "supervised machine learning"[MeSH Terms] OR "artificial intelligence"[Title/Abstract] OR "deep learning"[Title/Abstract] OR "supervised machine learning"[Title/Abstract]) | ("delivery of health care"[MeSH Terms] OR "health care"[Title/Abstract] OR "healthcare"[Title/Abstract] OR "medical"[Title/Abstract] OR "clinical"[Title/Abstract]) | ("Medical History Taking"[MeSH Terms] OR "anamnes*"[All Fields] OR "Triage"[MeSH Terms]) |
| **Cinahl** | (MH "Neural Networks (Computer)" OR MH "Artificial Intelligence" OR MH "Deep Learning" OR MH "Machine Learning" OR "artificial intelligence" OR "deep learning" OR "machine learning" OR "supervised machine learning") | (MH"health care" OR healthcare OR clinical OR medical) | (MH “Patient history taking” OR Anamnes* OR MH Triage) |
| **PsycINFO (ProQuest)** | MAINSUBJECT.EXACT("Deep Neural Networks") OR MAINSUBJECT.EXACT("Artificial Intelligence") OR MAINSUBJECT.EXACT("Machine Learning") OR noft("neural networks" OR "artificial intelligence" OR "deep learning" OR "machine learning" OR "supervised machine learning") | MAINSUBJECT.EXACT("Health Care Delivery") OR noft("health care" OR healthcare OR medical OR clinical) | MAINSUBJECT.EXACT("Patient History") OR noft( anamnes* OR triage) |
| **Scopus** | (TITLE-ABS-KEY ( "neural networks" OR "artificial intelligence" OR "deep learning" OR "machine learning" OR "supervised machine learning") | (TITLE-ABS-KEY("health care" OR healthcare OR medical OR clinical)) | (TITLE-ABS-KEY ("medical history taking" OR anamnes* OR triage)) |
| **Web of Science: Core Collection** | TS=("artificial intelligence" OR "deep learning" OR "neural networks" OR "machine learning" OR "supervised machine learning") | TS=("health care" OR healthcare OR clinical OR medical)) | TS=("medical history taking" OR anamnes* OR triage) |

Table 2. Search strings by database (PubMed, CINAHL, PsycINFO, Scopus, Web of Science Core Collection)

| **DATABASE** | **SEARCH STRING** |
| --- | --- |
| Pubmed (used)  *Filter by publication date 200101-220930* | (("neural networks, computer"[MeSH Terms:noexp] OR "artificial intelligence"[MeSH Terms:noexp] OR "deep learning"[MeSH Terms] OR "supervised machine learning"[MeSH Terms] OR "artificial intelligence"[Title/Abstract] OR "deep learning"[Title/Abstract] OR "supervised machine learning"[Title/Abstract]) AND ("delivery of health care"[MeSH Terms] OR "health care"[Title/Abstract] OR "healthcare"[Title/Abstract] OR "medical"[Title/Abstract] OR "clinical"[Title/Abstract])) AND ("Medical History Taking"[MeSH Terms] OR "anamnes*"[All Fields] OR "Triage"[MeSH Terms]) |
| Cinahl  (used)  *Filter by publication date 200101- 220930*  *“All results”* | (MH "Neural Networks (Computer)" OR MH "Artificial Intelligence" OR MH "Deep Learning" OR MH "Machine Learning" OR "artificial intelligence" OR "deep learning" OR "machine learning" OR "supervised machine learning") AND ("health care" OR healthcare OR clinical OR medical) AND (MH “Patient history taking” OR Anamnes* OR MH Triage)) |
| PsychINFO  (used)  *Filter by date 200101-220930* | noft("neural networks" OR "artificial intelligence" OR "deep learning" OR "machine learning" OR "supervised machine learning") AND noft("health care" OR healthcare OR medical OR clinical) AND noft(“medical history taking” OR “anamnes*” OR triage) |
| PsychINFO  (used)  *Filter by date 200101-220930* | (MAINSUBJECT.EXACT("Deep Neural Networks") OR MAINSUBJECT.EXACT("Artificial Intelligence") OR MAINSUBJECT.EXACT("Machine Learning") OR noft("neural networks" OR "artificial intelligence" OR "deep learning" OR "machine learning" OR "supervised machine learning")) AND (MAINSUBJECT.EXACT("Health Care Delivery") OR noft("health care" OR healthcare OR medical OR clinical)) AND (MAINSUBJECT.EXACT("Patient History") OR noft("medical history taking" OR anamnes* OR triage)) |
| Scopus  (used)  *Filter by publication date 2000-2022* | ( TITLE-ABS-KEY ( "neural networks" OR "artificial intelligence" OR "deep learning" OR "machine learning" OR "supervised machine learning" )) AND ( TITLE-ABS-KEY ( "health care" OR healthcare OR medical OR clinical )) AND ( TITLE-ABS-KEY ( "medical history taking" OR anamnes* OR triage )) |
| Web of Science  *Filter by publication date 200101-220930* | ((TS=("artificial intelligence" OR "deep learning" OR "neural networks" OR "machine learning" OR "supervised machine learning")) AND TS=("health care" OR healthcare OR clinical OR medical)) AND TS=("medical history taking" OR anamnes* OR triage) |

Table 3. Search results (number of hits) by search blocks and database (PubMed, CINAHL, PsycINFO, Scopus, Web of Science Core Collection).

| **Database** | **Pubmed** | **CINAHL (Ebsco)** | **PsycINFO (ProQuest)** | **Scopus** | **Web of Science Core Collection** |  |
| --- | --- | --- | --- | --- | --- | --- |
| **Date of search** | 221019 | 221019 | 221019 | 221019 | 221019 |  |
| **Filter by publication date** | 200101-220930 | 200101-220930 | 200101-220930 | 2000-2022 | 200101-220930 |  |
| **Search block** |  |  |  |  |  |  |
| Artificial intelligence (AI) | 115,879 | 24,928 | 66,787 | 1,122,310 | 420,323 |  |
| Healthcare | 4,865,214 | 2,226,856 | 1,239,580 | 10,334,387 | 4,488,217 |  |
| Medical history-taking and/or triage | 31,768 | 24,050 | 7,484 | 152,058 | 30,681 |  |
| Artificial intelligence (AI) **and** Healthcare | 32,743 | 12,477 | 13,406 | 149,238 | 51,985 |  |
| Artificial intelligence (AI) **and** Healthcare **and** Medical history-taking and/or triage | 172 | 124 | 57 | 1115 | 540 |  |
| **Total number of hits** | 172 | 124 | 57 | 1115 | 540 | 2008 |
| **Number of duplicates** |  |  |  |  |  | 760 |
| **Total sources retrieved from databases** |  |  |  |  |  | 1248 |
| **Included articles** |  |  |  |  |  | 86 |
