# Supplementary material for: Mapping and Summarizing the Research on AI Systems for Automating Medical History Taking and Triage: Scoping Review
Source: J Med Internet Res. 2025 Feb 6;27:e53741. doi: 10.2196/53741 (PMC11843066; doi:10.2196/53741)
Supplement: Multimedia Appendix 3 [file jmir_v27i1e53741_app3.docx]

**Multimedia Appendix 2**

Table 1. Characteristics of included studies (n=86).

| **Authors** | **Publication date** | **Title** | **Country** | **Study design** | **Clinical context** | **Sample population** | **Perspective described** | **Type of AI system** | **Task performed by AI system** | **TRL level 1-9** |
| --- | --- | --- | --- | --- | --- | --- | --- | --- | --- | --- |
| Abe et al[1] | 2022 | A Prehospital Triage System to Detect Traumatic Intracranial Hemorrhage Using Machine Learning Algorithms | Japan | Retrospective study | Emergency care | Patients | Researcher | Hybrid model | Recognition | 4 |
| Ahmad et al[2] | 2019 | A rapid triage test for active pulmonary tuberculosis in adult patients with persistent cough | US | Prospective and retrospective study | Primary care | Patients | Researcher | Statistical model | Forecasting | 5 |
| Ahmed et al[3] | 2022 | Accuracy of the Traditional COVID-19 Phone Triaging System and Phone Triage-Driven Deep Learning Model | Egypt | Retrospective study | Primary care | Patients | Researcher | Hybrid model | Forecasting | 4 |
| Annarumma et al[4] | 2019 | Automated Triaging of Adult Chest Radiographs with Deep Artificial Neural Networks | UK | Retrospective study | Radiology | Patients | Researcher | Hybrid model | Forecasting | 4 |
| Ayling et al[5] | 2021 | Use of ColonFlag score for prioritisation of endoscopy in colorectal cancer | UK | Retrospective study | Gastroenterology | Patients | Researcher | Hybrid model | Forecasting | 5 |
| Azeez et al[6] | 2013 | Comparison of adaptive neuro-fuzzy inference system and artificial neutral networks model to categorize patients in the emergency department | Malaysia | Retrospective study | Emergency care | Patients | Researcher | Hybrid model | Recognition | 4 |
| Azeez et al[7] | 2015 | Secondary triage classification using an ensemble random forest technique | Malaysia | Retrospective study | Emergency care | Patients | Researcher | Hybrid model | Recognition | 4 |
| Baker et al[8] | 2020 | A Comparison of Artificial Intelligence and Human Doctors for the Purpose of Triage and Diagnosis | UK | Prospective study | Primary care | Healthcare professionals | Researcher | Hybrid model | Recognition | 5 |
| Chang et al[9] | 2022 | Machine learning-based triage to identify low-severity patients with a short discharge length of stay in emergency department | China and Taiwan | Retrospective study | Emergency care | Patients | Researcher | Hybrid model | Forecasting | 5 |
| Choi et al[10] | 2019 | Machine Learning-Based Prediction of Korean Triage and Acuity Scale Level in Emergency Department Patients | Korea | Cross-sectional study | Emergency care | Patients | Researcher | Hybrid model | Forecasting | 4 |
| Dehghani Soufi et al [11] | 2018 | Decision support system for triage management: A hybrid approach using rule-based reasoning and fuzzy logic | Iran | Not specified | Emergency care | Patients | Researcher | Hybrid model | Forecasting | 4 |
| Delshad et al[12] | 2021 | Artificial Intelligence-Based Application Provides Accurate Medical Triage Advice When Compared to Consensus Decisions of Healthcare Providers | US | Not specified | Not specified | Healthcare professionals | Researcher | Hybrid model | Recognition | 5 |
| Dembrower et al[13] | 2020 | Effect of artificial intelligence-based triaging of breast cancer screening mammograms on cancer detection and radiologist workload: a retrospective simulation study | Sweden | Retrospective study | Radiology | Patients | Researcher | Hybrid model | Forecasting | 5 |
| Denecke et al[14] | 2018 | Self-Anamnesis with a Conversational User Interface: Concept and Usability Study | Switzerland | Not specified | Music therapy | Citizens | Citizen | Hybrid model | Interaction support | 3 |
| Duceau et al[15] | 2020 | Prehospital triage of acute aortic syndrome using a machine learning algorithm | France | Observational study | Emergency care | Patients | Researcher | Hybrid model | Forecasting | 4 |
| Dyer et al[16] | 2022 | Validation of an artificial intelligence solution for acute triage and rule-out normal of non-contrast CT head scans | UK, US and India | Retrospective study | Radiology | Patients | Researcher | Hybrid model | Recongition | 5 |
| Entezarjou et al [17] | 2020 | Human- Versus Machine Learning-Based Triage Using Digitalized Patient Histories in Primary Care: Comparative Study | Sweden | Not specified | Primary care | Healthcare professionals | Researcher | Hybrid model | Recongition | 4 |
| Fernandes et al[18] | 2020 | Risk of mortality and cardiopulmonary arrest in critical patients presenting to the emergency department using machine learning and natural language processing | Portugal and US | Not specified | Emergency care | Patients | Researcher | Hybrid model | Forecasting | 4 |
| Fernandes et al[19] | 2020 | Predicting intensive care unit admission among patients presenting to the emergency department using machine learning and natural language processing | Portugal | Not specified | Emergency care | Patients | Researcher | Statistical model | Forecasting | 4 |
| Fernandes et al[20] | 2020 | Clinical Decision Support Systems for Triage in the Emergency Department using Intelligent Systems: a Review | n/a | Literature review | Emergency care | n/a | Researcher | n/a | n/a | n/a |
| Gao et al[21] | 2022 | Developing and Validating an Emergency Triage Model Using Machine Learning Algorithms with Medical Big Data | China | Retrospective study | Emergency care | Patients | Researcher | Hybrid model | Forecasting | 4 |
| Giavina-Bianchi et al[22] | 2021 | Accuracy of Deep Neural Network in Triaging Common Skin Diseases of Primary Care Attention | Brazil | Not specified | Dermatology | Patients | Researcher | Hybrid model | Recognition | 4 |
| Goncharov et al[23] | 2021 | CT-Based COVID-19 triage: Deep multitask learning improves joint identification and severity quantification | Russia | Not specified | Radiology | Patients | Researcher | Hybrid model | Recognition | 4 |
| Goto et al[24] | 2019 | Machine Learning-Based Prediction of Clinical Outcomes for Children during Emergency Department Triage | US | Prognostic study | Emergency care | Patients | Researcher | Hybrid model | Forecasting | 4 |
| Gottliebsen & Petersson[25] | 2020 | Limited evidence of benefits of patient operated intelligent primary care triage tools: findings of a literature review | n/a | Literature review | Primary care | n/a | Researcher | n/a | n/a | n/a |
| Gross et al[26] | 2020 | Validity of the Work Assessment Triage Tool for Selecting Rehabilitation Interventions for Workers’ Compensation Claimants with Musculoskeletal Conditions | Canada | Population-based cohort study | Rehabilitation | Patients | Researcher | Hybrid model | Recognition | 5 |
| Harada et al[27] | 2021 | Efficacy of Artificial-Intelligence-Driven Differential-Diagnosis List on the Diagnostic Accuracy of Physicians: An Open-Label Randomized Controlled Study | Japan | Randomized controlled study | Not specified | Healthcare professionals | Researcher | Not specified | Not specified | 5 |
| Heydon et al[28] | 2020 | Prospective evaluation of an artificial intelligence-enabled algorithm for automated diabetic retinopathy screening of 30 000 patients | UK | Prospective study | Not specified | Patients | Researcher | Hybrid model | Recognition | 6 |
| Hwang & Lee[29] | 2022 | Machine learning-based prediction of critical illness in children visiting the emergency department | Korea | Cross-sectional study | Emergency care | Patients | Researcher | Hybrid model | Forecasting | 4 |
| Ivanov et al[30] | 2021 | Improving ED Emergency Severity Index Acuity Assignment Using Machine Learning and Clinical Natural Language Processing | US | Retrospective study | Emergency care | Patients | Researcher | Hybrid model | Forecasting | 4 |
| Jiang et al[31] | 2021 | Machine learning-based models to support decision-making in emergency department triage for patients with suspected cardiovascular disease | China | Cross-sectional study | Emergency care | Patients | Researcher | Hybrid model | Forecasting | 4 |
| Jordan et al[32] | 2022 | The Impact of Cultural Embeddedness on the Implementation of an Artificial Intelligence Program at Triage: A Qualitative Study | US | Exploratory qualitative study | Emergency care | Healthcare professionals | Healthcare professional | Not specified | Not specified | 9 |
| Joseph et al[33] | 2020 | Deep-learning approaches to identify critically Ill patients at emergency department triage using limited information | US | Retrospective study | Emergency care | Patients | Researcher | Hybrid model | Forecasting | 4 |
| Joudar et al[34] | 2022 | Triage and priority-based healthcare diagnosis using artificial intelligence for autism spectrum disorder and gene contribution: A systematic review | n/a | Literature review | Psychiatry | n/a | Researcher | n/a | n/a | n/a |
| Kawamura et al[35] | 2022 | Incidence of Diagnostic Errors Among Unexpectedly Hospitalized Patients Using an Automated Medical History–Taking System With a Differential Diagnosis Generator: Retrospective Observational Study | Japan | Retrospective study | Not specified | Patients | Researcher | Hybrid model | Interaction support | 8 |
| Kerr et al[36] | 2021 | Human vital sign determination using tactile sensing and fuzzy triage system | UK | Pilot study | Not specified | Non-patients | Researcher | Hybrid model | Recognition | 5 |
| Kim et al[37] | 2022 | An automated COVID-19 triage pipeline using artificial intelligence based on chest radiographs and clinical data | US | Retrospective study | Radiology | Patients | Researcher | Hybrid model | Forecasting | 4 |
| Kim et al[38] | 2018 | A data-driven artificial intelligence model for remote triage in the prehospital environment | US | Not specified | Pre-hospital care | Patients | Researcher | Hybrid model | Recognition | 4 |
| Kim et al[39] | 2021 | Optimal triage for covid-19 patients under limited health care resources with a parsimonious machine learning prediction model and threshold optimization using discrete-event simulation: Development study | Korea | Retrospective study | Not specified | Patients | Researcher | Hybrid model | Forecasting | 4 |
| Knitza et al[40] | 2022 | Machine learning-based improvement of an online rheumatology referral and triage system | Germany | Not specified | Rheumatology | Patients | Researcher | Hybrid model | Recognition | 4 |
| Kwon et al[41] | 2018 | Validation of deep-learning-based triage and acuity score using a large national dataset | Korea | Retrospective study | Emergency care | Patients | Researcher | Hybrid model | Forecasting | 5 |
| Kyono et al[42] | 2021 | Triage of 2D Mammographic Images Using Multi-view Multi-task Convolutional Neural Networks | US | Not specified | Radiology | Patients | Researcher | Hybrid model | Recognition | 4 |
| Larsson et al[43] | 2021 | The advanced machine learner XGBoost did not reduce prehospital trauma mistriage compared with logistic regression: a simulation study | US and Sweden | Simulation study | Pre-hospital care | n/a | Researcher | Hybrid model | Forecasting | 4 |
| Levin et al[44] | 2018 | Machine-Learning-Based Electronic Triage More Accurately Differentiates Patients With Respect to Clinical Outcomes Compared With the Emergency Severity Index | US | Retrospective study | Emergency care | Patients | Researcher | Hybrid model | Forecasting | 4 |
| Libório et al[45] | 2022 | Impact of a computer system as a triage tool in the management of pulmonary tuberculosis in a HIV reference center in Brazil | Brazil | Prospective study | Not specified | Patients | Researcher | Hybrid model | Recognition | 4 |
| Liu et al[46] | 2021 | Development and validation of a practical machine-learning triage algorithm for the detection of patients in need of critical care in the emergency department | China | Prospective study | Emergency care | Patients | Researcher | Hybrid model | Recognition | 4 |
| Love et al[47] | 2018 | Palpable Breast Lump Triage by Minimally Trained Operators in Mexico Using Computer-Assisted Diagnosis and Low-Cost Ultrasound | Mexico | Pilot study | Radiology | Healthcare professionals | Researcher | Hybrid model | Recognition | 5 |
| Lowres et al[48] | 2020 | Use of a Machine Learning Program to Correctly Triage Incoming Text Messaging Replies From a Cardiovascular Text–Based Secondary Prevention Program: Feasibility Study | Australia | Not specified | Cardiology | Patients | Researcher | Hybrid model | Recognition | 4 |
| Majidian et al[49] | 2022 | Artificial Intelligence in the Evaluation of Telemedicine Dermatology Patients | US | Not specified | Dermatology | Healthcare professionals | Researcher | Not specified | Recognition | 4 |
| Morrill et al[50] | 2022 | A Machine Learning Methodology for Identification and Triage of Heart Failure Exacerbations | US | Not specified | Cardiology | Healthcare professionals | Researcher | Hybrid model | Forecasting | 4 |
| Morse et al[51] | 2020 | Use Characteristics and Triage Acuity of a Digital Symptom Checker in a Large Integrated Health System: Population-Based Descriptive Study | US | Population-Based descriptive study | Not specified | Patients | Researcher | Not specified | Not specified | 9 |
| Nederpelt et al[52] | 2021 | Development of a field artificial intelligence triage tool: Confidence in the prediction of shock, transfusion, and definitive surgical therapy in patients with truncal gunshot wounds | US | Retrospective study | Surgery, trauma | Patients | Researcher | Hybrid model | Forecasting | 3 |
| Nsengiyumva et al[53] | 2021 | Triage of Persons With Tuberculosis Symptoms Using Artificial Intelligence-Based Chest Radiograph Interpretation: A Cost-Effectiveness Analysis | Pakistan | Prospective study | Radiology | Patients | Researcher | Not specified | Recognition | 5 |
| Qin et al[54] | 2021 | Tuberculosis detection from chest x-rays for triaging in a high tuberculosis-burden setting: an evaluation of five artificial intelligence algorithms | Bangladesh | Retrospective study | Radiology | Patients | Researcher | Hybrid model | Recognition | 5 |
| Raita et al[55] | 2019 | Emergency department triage prediction of clinical outcomes using machine learning models | US | Not specified | Emergency care | Patients | Researcher | Hybrid model | Forecasting | 4 |
| Sánchez-Salmerón et al[56] | 2022 | Machine learning methods applied to triage in emergency services: A systematic review | Spain | Literature review | Emergency care | n/a | Researcher | n/a | n/a | n/a |
| Sanders & Mann Iii | 2000 | Automated scoring of patient pain drawings using artificial neural networks: Efforts toward a low back pain triage application | US | Not specified | Not specified | Patients | Researcher | Hybrid model | Recognition | 3 |
| Scheder-Bieschin et al[57] | 2022 | Improving Emergency Department Patient-Physician Conversation Through an Artificial Intelligence Symptom-Taking Tool: Mixed Methods Pilot Observational Study | Germany | Pilot study | Emergency care | Patients | Healthcare professional and patient | Symbolic or knowledge-based model | Interaction support | 7 |
| Scheetz et al[58] | 2007 | Using crash scene variables to predict the need for trauma center care in older persons | US | Exploratory data mining study | Emergency care | Patients | Researcher | Symbolic or knowledge-based model | Forecasting | 4 |
| Senda et al[59] | 2022 | Development of practical triage methods for critical trauma patients: machine-learning algorithm for evaluating hybrid operation theatre entry of trauma patients (THETA) | Japan | Retrospective study | Surgery, trauma | Patients | Researcher | Hybrid model | Forecasting | 4 |
| Shazzadur Rahman et al[60] | 2019 | Modelling the impact of chest X-ray and alternative triage approaches prior to seeking a tuberculosis diagnosis | Brazil | Not specified | Not specified | Patients | Researcher | Hybrid model | Recognition | 4 |
| Shiraz et al[61] | 2022 | Cervical cell lift: A novel triage method for the spatial mapping and grading of precancerous cervical lesions | UK | Pilot study | Not specified | Patients | Researcher | Hybrid model | Recognition | 4 |
| Singh et al[62] | 2018 | Machine learning for psychiatric patient triaging: an investigation of cascading classifiers | US | Not specified | Psychiatry | Patients | Researcher | Hybrid model | Recognition | 4 |
| Smith et al[63] | 2006 | Guided self diagnosis: an innovative approach to triage for emergency dental care | Australia | Not specified | Emergency dental care | Patients | Researcher | Hybrid model | Recognition | 3 |
| Soltan et al[64] | 2022 | Real-world evaluation of rapid and laboratory-free COVID-19 triage for emergency care: external validation and pilot deployment of artificial intelligence driven screening | UK | Not specified | Emergency care | Patients | Researcher | Not specified | Forecasting | 7 |
| Spasic & Button[65] | 2020 | Patient triage by topic modeling of referral letters: Feasibility study | UK | Not specified | Primary care | Patients | Researcher | Statistical model | Forecasting | 4 |
| Swaminathan et al[66] | 2017 | A machine learning approach to triaging patients with chronic obstructive pulmonary disease | US | Not specified | Pulmonology | Healthcare professionals | Researcher | Hybrid model | Forecasting | 4 |
| Tadesse et al[67] | 2020 | Multi-Modal Diagnosis of Infectious Diseases in the Developing World | Vietnam | Not specified | Not specified | Patients | Researcher | Hybrid model | Forecasting | 4 |
| Tan et al[68] | 2019 | Triaging ophthalmology outpatient referrals with machine learning: A pilot study | Australia | Retrospective study | Ophthalmology | Patients | Researcher | Statistical model | Forecasting | 4 |
| Tao et al[69] | 2022 | Scrutinizing high-risk patients from ASC-US cytology via a deep learning model | China | Retrospective study | Gynaecology | Patients | Researcher | Hybrid model | Forecasting | 3 |
| Tsai et al[70] | 2022 | Development and Validation of an Artificial Intelligence Electrocardiogram Recommendation System in the Emergency Department | Taiwan | Retrospective study | Emergency care | Patients | Researcher | Hybrid | Forecasting | 4 |
| Vaghefi et al[71] | 2021 | THEIA™ development, and testing of artificial intelligence-based primary triage of diabetic retinopathy screening images in New Zealand | New Zealand | Retrospective study | Ophthalmology | Patients | Researcher | Hybrid model | Recognition | 5 |
| van de Leur et al[72] | 2020 | Automatic Triage of 12-Lead ECGs Using Deep Convolutional Neural Networks | Netherlands | Not specified | Cardiology | Patients | Researcher | Hybrid model | Recognition | 4 |
| Verburg et al[73] | 2022 | Deep Learning for Automated Triaging of 4581 Breast MRI Examinations from the DENSE Trial | Netherlands | Prospective study | Radiology | Patients | Researcher | Hybrid model | Recognition | 4 |
| Wang & Feng[74] | 2022 | ERNIE based intelligent triage system | China | Not specified | Not specified | Patients | Researcher | Hybrid model | Recognition | 3 |
| Wang et al[75] | 2020 | Deep learning-based triage and analysis of lesion burden for COVID-19: a retrospective study with external validation | China | Retrospective study | Radiology | Patients | Researcher | Statistical model | Recognition | 5 |
| Wee et al[76] | 2022 | Triaging Medical Referrals Based on Clinical Prioritisation Criteria Using Machine Learning Techniques | Australia | Not specified | Otorhinolaryngology | Patients | Researcher | Hybrid model | Recognition | 3 |
| Wolff et al[77] | 2019 | Setting up standards: A methodological proposal for pediatric Triage machine learning model construction based on clinical outcomes | Chile | Retrospective study | Emergency care | Patients | Researcher | Hybrid model | Forecasting | 4 |
| Xie et al[78] | 2021 | Development and Assessment of an Interpretable Machine Learning Triage Tool for Estimating Mortality after Emergency Admissions | Singapore | Retrospective study | Emergency care | Patients | Researcher | Hybrid model | Forecasting | 4 |
| Xiong et al[79] | 2021 | Integrating transportation data with emergency medical service records to improve triage decision of high-risk trauma patients | US | Not specified | Emergency care | Patients | Researcher | Hybrid model | Forecasting | 3 |
| Yala et al[80] | 2019 | A deep learning model to triage screening mammograms: A simulation study | US | Retrospective study | Radiology | Patients | Researcher | Hybrid model | Recognition | 4 |
| Yang et al[81] | 2022 | Clinical Trial Classification of SNS24 Calls with Neural Networks | Portugal | Not specified | Not specified | Patients | Researcher | Hybrid model | Recognition | 4 |
| Yao et al[82] | 2021 | A Novel Deep Learning-Based System for Triage in the Emergency Department Using Electronic Medical Records: Retrospective Cohort Study | US and Taiwan | Retrospective study | Emergency care | Patients | Researcher | Hybrid model | Forecasting | 5 |
| Yu et al[83] | 2020 | Machine learning and initial nursing assessment-based triage system for emergency department | Korea | Retrospective study | Emergency care | Patients | Researcher | Hybrid model | Forecasting | 4 |
| Zhong et al[84] | 2021 | Integrated medical resource consumption stratification in hospitalized patients: an Auto Triage Management model based on accurate risk, cost and length of stay prediction | China | Retrospective study | Not specified | Patients | Researcher | Statistical model | Forecasting | 5 |
| Zmiri et al[85] | 2010 | Classification of patients by severity grades during triage in the emergency department using data mining methods | Israel | Not specified | Emergency care | Patients | Researcher | Hybrid model | Recognition | 4 |

**References**

1. Abe D, Inaji M, Hase T, Takahashi S, Sakai R, Ayabe F, et al. A Prehospital Triage System to Detect Traumatic Intracranial Hemorrhage Using Machine Learning Algorithms. JAMA Network Open. 2022 2022;5(6):e2216393-e. PMID: rayyan-393739430. doi: doi:doi:10.1001/jamanetworkopen.2022.16393.

2. Ahmad R, Xie L, Pyle M, Suarez MF, Broger T, Steinberg D, et al. A rapid triage test for active pulmonary tuberculosis in adult patients with persistent cough. Science Translational Medicine. 2019 2019;11(515). PMID: rayyan-393739431. doi: doi:doi:10.1126/scitranslmed.aaw8287.

3. Ahmed MM, Sayed AM, Khafagy GM, El Sayed IT, Elkholy YS, Fares AH, et al. Accuracy of the Traditional COVID-19 Phone Triaging System and Phone Triage-Driven Deep Learning Model. Journal of Primary Care and Community Health. 2022 2022;13. PMID: rayyan-387471433. doi: doi:doi:10.1177/21501319221113544.

4. Annarumma M, Withey SJ, Bakewell RJ, Pesce E, Goh V, Montana G. Automated Triaging of Adult Chest Radiographs with Deep Artificial Neural Networks. Radiology. 2019 2019-4;291(1):196-202. PMID: rayyan-393739432. doi: doi:doi:10.1148/radiol.2018180921.

5. Ayling RM, Wong A, Cotter F. Use of ColonFlag score for prioritisation of endoscopy in colorectal cancer. BMJ Open Gastroenterology. 2021 2021;8(1). PMID: rayyan-387471434. doi: doi:doi:10.1136/bmjgast-2021-000639.

6. Azeez D, Ali MAM, Gan KB, Saiboon I. Comparison of adaptive neuro-fuzzy inference system and artificial neutral networks model to categorize patients in the emergency department. SpringerPlus. 2013 2013;2(1):1-10. PMID: rayyan-387471435. doi: doi:doi:10.1186/2193-1801-2-416.

7. Azeez D, Gan KB, Mohd Ali MA, Ismail MS. Secondary triage classification using an ensemble random forest technique. Technol Health Care. 2015 2015;23(4):419-28. PMID: rayyan-387471436. doi: doi:doi:10.3233/thc-150907.

8. Baker A, Perov Y, Middleton K, Baxter J, Mullarkey D, Sangar D, et al. A Comparison of Artificial Intelligence and Human Doctors for the Purpose of Triage and Diagnosis. Frontiers in Artificial Intelligence. 2020 2020;3. PMID: rayyan-387471437. doi: doi:doi:10.3389/frai.2020.543405.

9. Chang Y-H, Shih H-M, Wu J-E, Huang F-W, Chen W-K, Chen D-M, et al. Machine learning-based triage to identify low-severity patients with a short discharge length of stay in emergency department. BMC Emergency Medicine. 2022 2022;22(1):1-10. PMID: rayyan-393739434. doi: doi:doi:10.1186/s12873-022-00632-6.

10. Choi SW, Ko T, Hong KJ, Kim KH. Machine Learning-Based Prediction of Korean Triage and Acuity Scale Level in Emergency Department Patients. Healthcare Informatics Research. 2019 2019-10;25(4):305-12. PMID: rayyan-393739435. doi: doi:doi:10.4258/hir.2019.25.4.305.

11. Dehghani Soufi M, Samad-Soltani T, Shams Vahdati S, Rezaei-Hachesu P. Decision support system for triage management: A hybrid approach using rule-based reasoning and fuzzy logic. International Journal of Medical Informatics. 2018 2018;114:35-44. PMID: rayyan-387471444. doi: doi:doi:10.1016/j.ijmedinf.2018.03.008.

12. Delshad S, Dontaraju VS, Chengat V. Artificial Intelligence-Based Application Provides Accurate Medical Triage Advice When Compared to Consensus Decisions of Healthcare Providers. Cureus. 2021 2021-8;13(8). PMID: rayyan-387471446. doi: doi:doi:10.7759/cureus.16956.

13. Dembrower K, Wåhlin E, Liu Y, Salim M, Smith K, Lindholm P, et al. Effect of artificial intelligence-based triaging of breast cancer screening mammograms on cancer detection and radiologist workload: a retrospective simulation study. The Lancet Digital Health. 2020 2020;2(9):e468-e74. PMID: rayyan-393739436. doi: doi:doi:10.1016/S2589-7500(20)30185-0.

14. Denecke K, Hochreutener SL, Popel A, May R. Self-Anamnesis with a Conversational User Interface: Concept and Usability Study. Methods of Information in Medicine. 2018 2018-11;57(5):243-52. PMID: rayyan-387471447. doi: doi:doi:10.1055/s-0038-1675822.

15. Duceau B, Alsac JM, Bellenfant F, Mailloux A, Champigneulle B, Favé G, et al. Prehospital triage of acute aortic syndrome using a machine learning algorithm. British Journal of Surgery. 2020 2020;107(8):995-1003. PMID: rayyan-387471448. doi: doi:doi:10.1002/bjs.11442.

16. Dyer T, Chawda S, Alkilani R, Morgan TN, Hughes M, Rasalingham S. Validation of an artificial intelligence solution for acute triage and rule-out normal of non-contrast CT head scans. Neuroradiology. 2022 2022-4;64(4):735-43. PMID: rayyan-393739437. doi: doi:doi:10.1007/s00234-021-02826-4.

17. Entezarjou A, Bonamy AKE, Benjaminsson S, Herman P, Midlov P. Human- Versus Machine Learning-Based Triage Using Digitalized Patient Histories in Primary Care: Comparative Study. Jmir Medical Informatics. 2020 2020-9;8(9). PMID: rayyan-387471449. doi: doi:doi:10.2196/18930.

18. Fernandes M, Mendes R, Vieira SM, Leite F, Palos C, Johnson A, et al. Risk of mortality and cardiopulmonary arrest in critical patients presenting to the emergency department using machine learning and natural language processing. Plos One. 2020 2020-4;15(4). PMID: rayyan-387471451. doi: doi:doi:10.1371/journal.pone.0230876.

19. Fernandes M, Mendes R, Vieira SM, Leite F, Palos C, Johnson A, et al. Predicting intensive care unit admission among patients presenting to the emergency department using machine learning and natural language processing. PLoS ONE. 2020 2020;15(3). PMID: rayyan-387471452. doi: doi:doi:10.1371/journal.pone.0229331.

20. Fernandes M, Vieira SM, Leite F, Palos C, Finkelstein S, Sousa JMC. Clinical Decision Support Systems for Triage in the Emergency Department using Intelligent Systems: a Review. Artif Intell Med. 2020 2020-1;102:101762. PMID: rayyan-387471453. doi: doi:doi:10.1016/j.artmed.2019.101762.

21. Gao Z, Qi X, Zhang X, Gao X, He X, Guo S, et al. Developing and Validating an Emergency Triage Model Using Machine Learning Algorithms with Medical Big Data. Risk Management and Healthcare Policy. 2022 2022;15:1545-51. PMID: rayyan-387471454. doi: doi:doi:10.2147/RMHP.S355176.

22. Giavina-Bianchi M, Cordioli E, Santos APD. Accuracy of Deep Neural Network in Triaging Common Skin Diseases of Primary Care Attention. Frontiers in Medicine. 2021 2021;8. PMID: rayyan-387471457. doi: doi:doi:10.3389/fmed.2021.670300.

23. Goncharov M, Pisov M, Shevtsov A, Shirokikh B, Kurmukov A, Blokhin I, et al. CT-Based COVID-19 triage: Deep multitask learning improves joint identification and severity quantification. Med Image Anal. 2021 2021-7;71:102054. PMID: rayyan-393739441. doi: doi:doi:10.1016/j.media.2021.102054.

24. Goto T, Camargo CA, Jr., Faridi MK, Freishtat RJ, Hasegawa K. Machine Learning-Based Prediction of Clinical Outcomes for Children during Emergency Department Triage. JAMA Network Open. 2019 2019;2(1). PMID: rayyan-387471458. doi: doi:doi:10.1001/jamanetworkopen.2018.6937.

25. Gottliebsen K, Petersson G. Limited evidence of benefits of patient operated intelligent primary care triage tools: findings of a literature review. BMJ Health Care Inform. 2020 2020-5;27(1). PMID: rayyan-387471459. doi: doi:doi:10.1136/bmjhci-2019-100114.

26. Gross DP, Steenstra IA, Shaw W, Yousefi P, Bellinger C, Zaïane O. Validity of the Work Assessment Triage Tool for Selecting Rehabilitation Interventions for Workers' Compensation Claimants with Musculoskeletal Conditions. J Occup Rehabil. 2020 Sep;30(3):318-30. PMID: 31267266. doi: 10.1007/s10926-019-09843-4.

27. Harada Y, Katsukura S, Kawamura R, Shimizu T. Efficacy of Artificial-Intelligence-Driven Differential-Diagnosis List on the Diagnostic Accuracy of Physicians: An Open-Label Randomized Controlled Study. Int J Environ Res Public Health. 2021 2021-2-21;18(4). PMID: rayyan-387471461. doi: doi:doi:10.3390/ijerph18042086.

28. Heydon P, Egan C, Bolter L, Chambers R, Anderson J, Aldington S, et al. Prospective evaluation of an artificial intelligence-enabled algorithm for automated diabetic retinopathy screening of 30 000 patients. British Journal of Ophthalmology. 2021 2021;105(5):723-8. PMID: rayyan-393739442. doi: doi:doi:10.1136/bjophthalmol-2020-316594.

29. Hwang S, Lee B. Machine learning-based prediction of critical illness in children visiting the emergency department. Plos One. 2022 2022-2;17(2). PMID: rayyan-393739443. doi: doi:doi:10.1371/journal.pone.0264184.

30. Ivanov O, Wolf L, Brecher D, Lewis E, Masek K, Montgomery K, et al. Improving ED Emergency Severity Index Acuity Assignment Using Machine Learning and Clinical Natural Language Processing. Journal of Emergency Nursing. 2021 2021;47(2):265-. PMID: rayyan-387471465. doi: doi:doi:10.1016/j.jen.2020.11.001.

31. Jiang H, Mao H, Lu H, Lin P, Garry W, Lu H, et al. Machine learning-based models to support decision-making in emergency department triage for patients with suspected cardiovascular disease. International Journal of Medical Informatics. 2021 2021;145. PMID: rayyan-387471466. doi: doi:doi:10.1016/j.ijmedinf.2020.104326.

32. Jordan M, Hauser J, Cota S, Li H, Wolf L. The Impact of Cultural Embeddedness on the Implementation of an Artificial Intelligence Program at Triage: A Qualitative Study. Journal of Transcultural Nursing. 2022 2022. PMID: rayyan-387471467. doi: doi:doi:10.1177/10436596221129226.

33. Joseph JW, Leventhal EL, Grossestreuer AV, Wong ML, Joseph LJ, Nathanson LA, et al. Deep-learning approaches to identify critically Ill patients at emergency department triage using limited information. Journal of the American College of Emergency Physicians Open. 2020 2020-10;1(5):773-81. PMID: rayyan-393739444. doi: doi:doi:10.1002/emp2.12218.

34. Joudar SS, Albahri AS, Hamid RA. Triage and priority-based healthcare diagnosis using artificial intelligence for autism spectrum disorder and gene contribution: A systematic review. Comput Biol Med. 2022 2022-7;146:105553. PMID: rayyan-387471468. doi: doi:doi:10.1016/j.compbiomed.2022.105553.

35. Kawamura R, Harada Y, Sugimoto S, Nagase Y, Katsukura S, Shimizu T. Incidence of Diagnostic Errors among Unexpectedly Hospitalized Patients Using an Automated Medical History-Taking System with a Differential Diagnosis Generator: Retrospective Observational Study. JMIR Medical Informatics. 2022 2022;10(1). PMID: rayyan-387471469. doi: doi:doi:10.2196/35225.

36. Kerr E, McGinnity TM, Coleman S, Shepherd A. Human vital sign determination using tactile sensing and fuzzy triage system. Expert Systems with Applications. 2021 2021;175. PMID: rayyan-387471470. doi: doi:doi:10.1016/j.eswa.2021.114781.

37. Kim CK, Choi JW, Jiao Z, Wang D, Wu J, Yi TY, et al. An automated COVID-19 triage pipeline using artificial intelligence based on chest radiographs and clinical data. npj Digital Medicine. 2022 2022;5(1). PMID: rayyan-393739448. doi: doi:doi:10.1038/s41746-021-00546-w.

38. Kim D, You S, So S, Lee J, Yook S, Jang DP, et al. A data-driven artificial intelligence model for remote triage in the prehospital environment. PLoS One. 2018;13(10):e0206006. PMID: 30352077. doi: 10.1371/journal.pone.0206006.

39. Kim J, Lim H, Ahn JH, Lee KH, Lee KS, Koo KC. Optimal triage for covid-19 patients under limited health care resources with a parsimonious machine learning prediction model and threshold optimization using discrete-event simulation: Development study. JMIR Medical Informatics. 2021 2021;9(11). PMID: rayyan-387471473. doi: doi:doi:10.2196/32726.

40. Knitza J, Janousek L, Kluge F, von der Decken CB, Kleinert S, Vorbrüggen W, et al. Machine learning-based improvement of an online rheumatology referral and triage system. Frontiers in Medicine. 2022 2022;9. PMID: rayyan-393739451. doi: doi:doi:10.3389/fmed.2022.954056.

41. Kwon JM, Lee Y, Lee Y, Lee S, Park H, Park J. Validation of deep-learning-based triage and acuity score using a large national dataset. PLoS ONE. 2018 2018;13(10). PMID: rayyan-387471476. doi: doi:doi:10.1371/journal.pone.0205836.

42. Kyono T, Gilbert FJ, Van Der Schaar M. Triage of 2D Mammographic Images Using Multi-view Multi-task Convolutional Neural Networks. ACM Transactions on Computing for Healthcare. 2021 2021;2(3). PMID: rayyan-393739454. doi: doi:doi:10.1145/3453166.

43. Larsson A, Berg J, Gellerfors M, Warnberg MG. The advanced machine learner XGBoost did not reduce prehospital trauma mistriage compared with logistic regression: a simulation study. Bmc Medical Informatics and Decision Making. 2021 2021-6;21(1). PMID: rayyan-387471478. doi: doi:doi:10.1186/s12911-021-01558-y.

44. Levin S, Toerper M, Hamrock E, Hinson JS, Barnes S, Gardner H, et al. Machine-Learning-Based Electronic Triage More Accurately Differentiates Patients With Respect to Clinical Outcomes Compared With the Emergency Severity Index. Annals of Emergency Medicine. 2018 2018;71(5):565-74.e2. PMID: rayyan-387471480. doi: doi:doi:10.1016/j.annemergmed.2017.08.005.

45. Libório MP, Kritski A, Almeida IN, Miranda PFC, Mesquita JRL, Mota RMS, et al. Impact of a computer system as a triage tool in the management of pulmonary tuberculosis in a HIV reference center in Brazil. Rev Soc Bras Med Trop. 2022;55:e0451. PMID: 35946632. doi: 10.1590/0037-8682-0451-20.

46. Liu Y, Gao J, Liu J, Walline JH, Liu X, Zhang T, et al. Development and validation of a practical machine-learning triage algorithm for the detection of patients in need of critical care in the emergency department. Scientific Reports. 2021 2021;11(1). PMID: rayyan-387471484. doi: doi:doi:10.1038/s41598-021-03104-2.

47. Love SM, Berg WA, Podilchuk C, López Aldrete AL, Gaxiola Mascareño AP, Pathicherikollamparambil K, et al. Palpable Breast Lump Triage by Minimally Trained Operators in Mexico Using Computer-Assisted Diagnosis and Low-Cost Ultrasound. J Glob Oncol. 2018 2018-8;4:1-9. PMID: rayyan-393739463. doi: doi:doi:10.1200/jgo.17.00222.

48. Lowres N, Duckworth A, Redfern J, Thiagalingam A, Chow CK. Use of a Machine Learning Program to Correctly Triage Incoming Text Messaging Replies From a Cardiovascular Text-Based Secondary Prevention Program: Feasibility Study. JMIR Mhealth Uhealth. 2020 Jun 16;8(6):e19200. PMID: 32543439. doi: 10.2196/19200.

49. Majidian M, Tejani I, Jarmain T, Kellett L, Moy R. Artificial Intelligence in the Evaluation of Telemedicine Dermatology Patients. J Drugs Dermatol. 2022 Feb 1;21(2):191-4. PMID: 35133107. doi: 10.36849/jdd.6277.

50. Morrill J, Qirko K, Kelly J, Ambrosy A, Toro B, Smith T, et al. A Machine Learning Methodology for Identification and Triage of Heart Failure Exacerbations. Journal of Cardiovascular Translational Research. 2022 2022-2;15(1):103-15. PMID: rayyan-387471488. doi: doi:doi:10.1007/s12265-021-10151-7.

51. Morse KE, Ostberg NP, Jones VG, Chan AS. Use Characteristics and Triage Acuity of a Digital Symptom Checker in a Large Integrated Health System: Population-Based Descriptive Study. J Med Internet Res. 2020 2020-11-30;22(11):e20549. PMID: rayyan-387471489. doi: doi:doi:10.2196/20549.

52. Nederpelt CJ, Mokhtari AK, Alser O, Tsiligkaridis T, Roberts J, Cha M, et al. Development of a field artificial intelligence triage tool: Confidence in the prediction of shock, transfusion, and definitive surgical therapy in patients with truncal gunshot wounds. Journal of Trauma & Acute Care Surgery. 2021 2021;90(6):1054-60. PMID: rayyan-387471490. doi: doi:doi:10.1097/TA.0000000000003155.

53. Nsengiyumva NP, Hussain H, Oxlade O, Majidulla A, Nazish A, Khan AJ, et al. Triage of Persons With Tuberculosis Symptoms Using Artificial Intelligence-Based Chest Radiograph Interpretation: A Cost-Effectiveness Analysis. Open Forum Infectious Diseases. 2021 2021-12;8(12). PMID: rayyan-387471491. doi: doi:doi:10.1093/ofid/ofab567.

54. Qin ZZ, Ahmed S, Sarker MS, Paul K, Adel ASS, Naheyan T, et al. Tuberculosis detection from chest x-rays for triaging in a high tuberculosis-burden setting: an evaluation of five artificial intelligence algorithms. The Lancet Digital Health. 2021 2021;3(9):e543-e54. PMID: rayyan-393739477. doi: doi:doi:10.1016/S2589-7500(21)00116-3.

55. Raita Y, Goto T, Faridi MK, Brown DFM, Camargo CA, Jr., Hasegawa K. Emergency department triage prediction of clinical outcomes using machine learning models. Critical Care. 2019 2019;23(1). PMID: rayyan-387471494. doi: doi:doi:10.1186/s13054-019-2351-7.

56. Sánchez-Salmerón R, Gómez-Urquiza JL, Albendín-García L, Correa-Rodríguez M, Martos-Cabrera MB, Velando-Soriano A, et al. Machine learning methods applied to triage in emergency services: A systematic review. International Emergency Nursing. 2022 2022;60. PMID: rayyan-387471499. doi: doi:doi:10.1016/j.ienj.2021.101109.

57. Scheder-Bieschin J, Blümke B, de Buijzer E, Cotte F, Echterdiek F, Nacsa J, et al. Improving Emergency Department Patient-Physician Conversation Through an Artificial Intelligence Symptom-Taking Tool: Mixed Methods Pilot Observational Study. JMIR Form Res. 2022 2022-2-7;6(2):e28199. PMID: rayyan-387471501. doi: doi:doi:10.2196/28199.

58. Scheetz LJ, Zhang J, Kolassa JE. Using crash scene variables to predict the need for trauma center care in older persons. Research in Nursing and Health. 2007 2007;30(4):399-412. PMID: rayyan-387471502. doi: doi:doi:10.1002/nur.20203.

59. Senda A, Endo A, Kinoshita T, Otomo Y. Development of practical triage methods for critical trauma patients: machine-learning algorithm for evaluating hybrid operation theatre entry of trauma patients (THETA). European Journal of Trauma and Emergency Surgery. 2022 2022. PMID: rayyan-387471503. doi: doi:doi:10.1007/s00068-022-02002-0.

60. Shazzadur Rahman AAM, Langley I, Galliez R, Kritski A, Tomeny E, Squire SB. Modelling the impact of chest X-ray and alternative triage approaches prior to seeking a tuberculosis diagnosis. BMC Infectious Diseases. 2019 2019;19(1). PMID: rayyan-387471504. doi: doi:doi:10.1186/s12879-019-3684-1.

61. Shiraz A, Egawa N, Pelt DM, Crawford R, Nicholas AK, Romashova V, et al. Cervical cell lift: A novel triage method for the spatial mapping and grading of precancerous cervical lesions. eBioMedicine. 2022 2022;82. PMID: rayyan-387471506. doi: doi:doi:10.1016/j.ebiom.2022.104157.

62. Singh VK, Shrivastava U, Bouayad L, Padmanabhan B, Ialynytchev A, Schultz SK. Machine learning for psychiatric patient triaging: an investigation of cascading classifiers. Journal of the American Medical Informatics Association. 2018 2018;25(11):1481-7. PMID: rayyan-387471507. doi: doi:doi:10.1093/jamia/ocy109.

63. Smith K, Clark A, Dyson K, Kruger E, Lejmanoski L, Russell A, et al. Guided self diagnosis: an innovative approach to triage for emergency dental care. Aust Dent J. 2006 2006-3;51(1):11-5. PMID: rayyan-387471508. doi: doi:doi:10.1111/j.1834-7819.2006.tb00394.x.

64. Soltan AAS, Yang J, Pattanshetty R, Novak A, Yang Y, Rohanian O, et al. Real-world evaluation of rapid and laboratory-free COVID-19 triage for emergency care: external validation and pilot deployment of artificial intelligence driven screening. Lancet Digit Health. 2022 2022-4;4(4):e266-e78. PMID: rayyan-387471509. doi: doi:doi:10.1016/s2589-7500(21)00272-7.

65. Spasic I, Button K. Patient triage by topic modeling of referral letters: Feasibility study. JMIR Medical Informatics. 2020 2020;8(11). PMID: rayyan-387471510. doi: doi:doi:10.2196/21252.

66. Swaminathan S, Qirko K, Smith T, Corcoran E, Wysham NG, Bazaz G, et al. A machine learning approach to triaging patients with chronic obstructive pulmonary disease. PLoS ONE. 2017 2017;12(11). PMID: rayyan-387471511. doi: doi:doi:10.1371/journal.pone.0188532.

67. Tadesse GA, Javed H, Thanh NLN, Thi HDH, Tan LV, Thwaites L, et al. Multi-Modal Diagnosis of Infectious Diseases in the Developing World. IEEE Journal of Biomedical and Health Informatics. 2020 2020;24(7):2131-41. PMID: rayyan-387471512. doi: doi:doi:10.1109/JBHI.2019.2959839.

68. Tan Y, Bacchi S, Casson RJ, Selva D, Chan W. Triaging ophthalmology outpatient referrals with machine learning: A pilot study. Clinical and Experimental Ophthalmology. 2020 2020;48(2):169-73. PMID: rayyan-393739491. doi: doi:doi:10.1111/ceo.13666.

69. Tao X, Chu X, Guo B, Pan Q, Ji S, Lou W, et al. Scrutinizing high-risk patients from ASC-US cytology via a deep learning model. Cancer Cytopathology. 2022 2022;130(6):407-14. PMID: rayyan-393739496. doi: doi:doi:10.1002/cncy.22560.

70. Tsai DA, Tsai SH, Chiang HH, Lee CC, Chen O. Development and Validation of an Artificial Intelligence Electrocardiogram Recommendation System in the Emergency Department. Journal of Personalized Medicine. 2022 2022-5;12(5). PMID: rayyan-387471513. doi: doi:doi:10.3390/jpm12050700.

71. Vaghefi E, Yang S, Xie L, Hill S, Schmiedel O, Murphy R, et al. THEIA™ development, and testing of artificial intelligence-based primary triage of diabetic retinopathy screening images in New Zealand. Diabet Med. 2021 2021-4;38(4):e14386. PMID: rayyan-393739502. doi: doi:doi:10.1111/dme.14386.

72. van de Leur RR, Blom LJ, Gavves E, Hof IE, van der Heijden JF, Clappers NC, et al. Automatic Triage of 12-Lead ECGs Using Deep Convolutional Neural Networks. Journal of the American Heart Association. 2020 2020-5;9(10). PMID: rayyan-393739507. doi: doi:doi:10.1161/jaha.119.015138.

73. Verburg E, Van Gils CH, Van Der Velden BHM, Bakker MF, Pijnappel RM, Veldhuis WB, et al. Deep Learning for Automated Triaging of 4581 Breast MRI Examinations from the DENSE Trial. Radiology. 2022 2022;302(1):29-36. PMID: rayyan-393739509. doi: doi:doi:10.1148/radiol.2021203960.

74. Wang C, Feng F. ERNIE based intelligent triage system. Journal of Intelligent and Fuzzy Systems. 2022 2022;43(4):5013-22. PMID: rayyan-387471514. doi: doi:doi:10.3233/JIFS-212140.

75. Wang M, Xia C, Huang L, Xu S, Qin C, Liu J, et al. Deep learning-based triage and analysis of lesion burden for COVID-19: a retrospective study with external validation. Lancet Digit Health. 2020 2020-10;2(10):e506-e15. PMID: rayyan-387471515. doi: doi:doi:10.1016/s2589-7500(20)30199-0.

76. Wee CK, Zhou X, Sun R, Gururajan R, Tao X, Li Y, et al. Triaging Medical Referrals Based on Clinical Prioritisation Criteria Using Machine Learning Techniques. International Journal of Environmental Research and Public Health. 2022 2022;19(12). PMID: rayyan-393739514. doi: doi:doi:10.3390/ijerph19127384.

77. Wolff P, Rios SA, Grana M. Setting up standards: A methodological proposal for pediatric Triage machine learning model construction based on clinical outcomes. Expert Systems with Applications. 2019 2019-12;138. PMID: rayyan-387471516. doi: doi:doi:10.1016/j.eswa.2019.07.005.

78. Xie F, Ong MEH, Liew JNMH, Tan KBK, Ho AFW, Nadarajan GD, et al. Development and Assessment of an Interpretable Machine Learning Triage Tool for Estimating Mortality after Emergency Admissions. JAMA Network Open. 2021 2021;4(8). PMID: rayyan-387471518. doi: doi:doi:10.1001/jamanetworkopen.2021.18467.

79. Xiong C, Yang M, Kozar R, Zhang L. Integrating transportation data with emergency medical service records to improve triage decision of high-risk trauma patients. Journal of Transport & Health. 2021 2021/09/01/;22:101106. doi: <https://doi.org/10.1016/j.jth.2021.101106>.

80. Yala A, Schuster T, Miles R, Barzilay R, Lehman C. A deep learning model to triage screening mammograms: A simulation study. Radiology. 2019 2019;293(1):38-46. PMID: rayyan-393739518. doi: doi:doi:10.1148/radiol.2019182908.

81. Yang H, Goncalves T, Quaresma P, Vieira R, Veladas R, Pinto CS, et al. Clinical Trial Classification of SNS24 Calls with Neural Networks. Future Internet. 2022 2022-5;14(5). PMID: rayyan-387471519. doi: doi:doi:10.3390/fi14050130.

82. Yao LH, Leung KC, Tsai CL, Huang CH, Fu LC. A Novel Deep Learning-Based System for Triage in the Emergency Department Using Electronic Medical Records: Retrospective Cohort Study. J Med Internet Res. 2021 2021-12-27;23(12):e27008. PMID: rayyan-387471520. doi: doi:doi:10.2196/27008.

83. Yu JY, Jeong GY, Jeong OS, Chang DK, Cha WC. Machine learning and initial nursing assessment-based triage system for emergency department. Healthcare Informatics Research. 2020 2020;26(1):13-9. PMID: rayyan-387471522. doi: doi:doi:10.4258/hir.2020.26.1.13.

84. Zhong Q, Li Z, Wang W, Zhang L, He K. Integrated medical resource consumption stratification in hospitalized patients: an Auto Triage Management model based on accurate risk, cost and length of stay prediction. Sci China Life Sci. 2022 2022-5;65(5):988-99. PMID: rayyan-387471523. doi: doi:doi:10.1007/s11427-021-1987-5.

85. Zmiri D, Shahar Y, Taieb-Maimon M. Classification of patients by severity grades during triage in the emergency department using data mining methods. Journal of Evaluation in Clinical Practice. 2012 2012;18(2):378-88. PMID: rayyan-387471524. doi: doi:doi:10.1111/j.1365-2753.2010.01592.x.
